# Imine Reductase Based All-Enzyme Hydrogel with Intrinsic Cofactor Regeneration for Flow Biocatalysis

**DOI:** 10.3390/mi10110783

**Published:** 2019-11-15

**Authors:** Patrick Bitterwolf, Felix Ott, Kersten S. Rabe, Christof M. Niemeyer

**Affiliations:** Institute for Biological Interfaces (IBG1), Karlsruhe Institute of Technology (KIT), 76131 Karlsruhe, Germany; patrick.bitterwolf@kit.edu (P.B.); felix.ott@student.kit.edu (F.O.); kersten.rabe@kit.edu (K.S.R.)

**Keywords:** enzyme immobilization, continuous, biocatalysis, imine reductase, microreactor

## Abstract

All-enzyme hydrogels are biocatalytic materials, with which various enzymes can be immobilized in microreactors in a simple, mild, and efficient manner to be used for continuous flow processes. Here we present the construction and application of a cofactor regenerating hydrogel based on the imine reductase GF3546 from *Streptomyces* sp. combined with the cofactor regenerating glucose-1-dehydrogenase from *Bacillus subtilis*. The resulting hydrogel materials were characterized in terms of binding kinetics and viscoelastic properties. The materials were formed by rapid covalent crosslinking in less than 5 min, and they showed a typical mesh size of 67 ± 2 nm. The gels were applied for continuous flow biocatalysis. In a microfluidic reactor setup, the hydrogels showed excellent conversions of imines to amines for up to 40 h in continuous flow mode. Variation of flow rates led to a process where the gels showed a maximum space-time-yield of 150 g·(L·day)^−1^ at 100 μL/min.

## 1. Introduction

Biocatalysis is emerging as a powerful approach for the production of active pharmaceutical ingredients (APIs) [1]. Based on the established “flow chemistry”, much effort is currently being devoted to transferring the concept machine-assisted modular chemical synthesis in miniaturized reactors to continuous and compartmentalized biocatalysis [2,3,4,5,6]. Compared to batch processes in conventional reaction vessels, microreactors offer large surface-to-volume ratios due to their dimensions in the sub-millimeter range, resulting in high heat transfer rates, fast diffusion processes, and laminar flow profiles. Furthermore, operation of microreactors under continuous flow allows sequential compartmentalization of complex multi-step reactions [5,7,8,9]. For example, the improved mass transport between individual compartments simplifies the addition of substrates, the separation of side products, and the avoidance of undesired crosstalk between various reaction steps [10,11]. Furthermore, this approach opens up numerous possibilities for the integration of automated in-line analysis, purification, and downstream processing.

Various strategies for the immobilization of biocatalysts are being explored to improve the stability of enzymes under process conditions and to reduce the total amount of catalyst or even enable its reuse [11,12]. Conventional methods for enzyme immobilization are based on chemical anchoring [13,14] or crosslinking of proteins [15], either directly on the reactor surface or on (porous) carrier materials. In fact, the use of carrier materials represents a compromise to increase the effective surface area for immobilization. However, this also inevitably reduces space-time yields, as the carrier material occupies a significant space in the reactor volume. To avoid this problem, we have recently developed all-enzyme hydrogels that allow the entire reactor room to be filled with biocatalytic material. By using the SpyCatcher/SpyTag system [16], either an (*R*)- or an (*S*)-selective alcohol dehydrogenase could be combined with a nicotinamide adenine dinucleotide phosphate (NADPH)-regenerating enzyme in a stoichiometrically defined manner [17,18]. The enzymes were genetically fused with the SpyTag peptide or the SpyCatcher protein domain to enable spontaneous covalent linkage via isopeptide bonds under physiological conditions. In contrast to conventional enzyme cross-linking methods, realized for instance in so-called cross-linked enzyme aggregates (CLEA) [15], this allows for a specific and mild immobilization with precise control over enzyme stoichiometry. The resulting porous hydrogel networks could conveniently be immobilized in microfluidic bioreactors, wherein they showed excellent performance in biocatalytic transformations [17,18].

To demonstrate the scope of this all enzyme hydrogel concept and to open it for further biocatalytic transformations, we report here on a novel hydrogel based on the NADPH dependent (*S*)-selective dimeric imine reductase (IRED) GF3546 from *Streptomyces* sp. [19] in combination with the NADPH cofactor regenerating homotetrameric glucose-1-dehydrogenase from *Bacillus subtilis*. IREDs, as well as other reductive aminases have attracted much attention in recent years because of their ability to generate optically active amines, which are key structural motifs in APIs [20,21]. For industrial purposes it is convenient to work with cell-free or immobilized enzymes to avoid cross-reactivities and, especially in the case of cofactor dependent enzymes, to gain control over economically attractive regeneration systems. However, only few examples of IREDs immobilized on carrier materials have been reported so far and none have been used for continuous flow biocatalysis [22,23].

## 2. Materials and Methods

All chemicals were purchased from Sigma Aldrich (St. Louis, MI, USA) or VWR (Radnor, PA, USA) if not stated otherwise. The gene encoding the imine reductase GF3546-ST was synthesized via “GeneArt Gene Synthesis” (Thermo Scientific, Waltham, MA, USA).

### 2.1. Plasmid Constructions

Unless stated otherwise, the genetic construction was carried out using the isothermal recombination as described by Gibson et al. utilizing oligonucleotide primers with 20–30 bp homologous overlaps [24]. Subsequently the reaction mixtures were DpnI treated to remove any remaining vector and transformed into *Escherichia coli* DH5α cells. Plasmids were purified using a ZR Plasmid Miniprep-Classic kit (Zymo Research, Freiburg, Germany) and commercial sequencing was used to verify the sequences (LGC genomics, Berlin, Germany).

The homotetrameric GDH-SC (41 kDa per monomer) was cloned as previously described [18]. For the cloning of the dimeric GF3546-ST (33 kDa per monomer) the following primers were used:

KSR227-for: TGAGATCCGGCTGCTAACAAAGCCC
KSR65-rev: ATGTATATCTCCTTCTTAAAGTTAAACAAAATTATTTCTAGAGGGG

Both enzymes were fused with a hexahistidin tag at the N- (GF3546-ST) or C- (BsGDH-SC) terminus, respectively. Additionally, linker sequences (GGGGS) were inserted between the ST/SC tags and the enzyme sequences.

### 2.2. Proteinexpression and Purifcation

For the expression of the proteins a *E. coli* BL21 (DE3) cell line was used and the corresponding vectors were transformed via heat shock transformation. After overnight culturing at 37 °C on lysogeny broth (LB) agar-plates supplemented with 100 μg/mL ampicillin, successful transformed clones were selected and transferred to liquid LB_amp_ medium and incubated for 14–18 h at 37 °C and 180 rpm.

A total of 2 L ampicillin-containing LB-medium was inoculated 1:20 with overnight culture. The cultures were incubated at 37 °C, 180 rpm until an OD600 of 0.6 was reached. Subsequently the temperature was set to 25 °C and the culture was induced with 0.1 mM isopropyl β-D-1-thiogalactopyranoside (IPTG). The cultures were incubated again for 16 h. The cultures were pooled, and cells were harvested by centrifugation (10,000× *g*, 10 min, 4 °C). The cells were resuspended using 60 mL of 50 mM NaH_2_PO_4_, 300 mM NaCl, 10 mM Imidazole buffer at pH 8.0. The cell lysate was centrifuged (45,000× *g*, 1 h, 4 °C) after disruption via ultrasonication and the supernatant was filtered through a 0.45 μm Durapore polyvinylidene fluoride (PVDF) membrane (Steriflip, Millipore, Burlington, MA, USA) and loaded on two HisTrap FF (5 mL) Ni-NTA columns (GE Healthcare, Freiburg, Germany) connected in series and mounted on an Äkta Pure liquid chromatography system (GE Healthcare, Germany). The columns were washed with 100 mL buffer A and the 6xHis-tagged proteins were eluted using a gradient from 100% buffer A to 100% buffer B (50 mM NaH_2_PO_4_, 300 mM NaCl, 500 mM Imidazole, pH 8.0) over a volume of 200 mL. Subsequently, the buffer was exchanged to KP_i_-Mg (100 mM K_2_HPO_4_/KH_2_PO_4_ pH 7.5, 1 mM MgCl_2_) using Vivaspin 10.000 MWCO (GE Healthcare).

To characterize the recombinant, purified proteins, samples were typically analyzed by standard discontinuous sodium dodecyl sulfate (SDS)-polyacrylamide Laemmli-midi-gels. The bands were visualized by coomassie staining and compared to a protein marker (Color Prestained Protein Standard, Broad Range (New England Biolabs)). Ultraviolet-visible (UV-Vis) spectroscopy (Eppendorf AG, Hamburg, Germany) was used to measure the protein concentration at 280 nm. The theoretical molar extinction coefficient was calculated via the Geneious software version 9.1.3 (Biomatters Ltd., Aukland, New Zealand) [25].

### 2.3. SDS Polyacrylamide Gel Electrophoresis Analysis

For SDS polyacrylamide gel electrophoresis (SDS-PAGE) analyses samples were mixed with 4× SDS-PAGE loading buffer (200 mM Tris-Cl pH 6.8; 400 mM DTT; 8% SDS; 0.4% bromophenol blue; 40% glycerol) to 1× concentration. The samples were subsequently boiled at 95 °C for 5 min and loaded onto a 1 mm thick SDS-PAGE gel containing 12% acrylamide. The gels were run at 100–120 V and coomassie stained. As a molecular weight marker Color Prestained Protein Standard, Broad Range (New England Biolabs, Beverly, MA, USA) was used.

### 2.4. Dynamic Light Scattering Measurements

The changes in hydrodynamic diameter upon polymerization was determined over time by dynamic light scattering (DLS) analyses, using the Nano-Series ZetaSizer (Zetasizer Nano ZSP, Malvern Instruments, Malvern, UK), equipped with a He-Ne-Laser (633 nm). Prior to DLS measurement, all protein solutions were preincubated in a thermoshaker for 5 min at the temperature of the following measurement and 500 rpm. DLS measurements were carried out at 30 °C over 2 h with individual measurements every 90 s.

### 2.5. Microrheology Measurements

Microrheology measurements based on multiple particle tracking (MPT) analyses were carried out as previously described [17]. In brief, hydrogels were prepared and supplemented with 0.2 mg/mL “dragon” green fluorescent polystyrene microspheres (200 nm diameter; Bangs Laboratories, Fishers, IN, USA). These gels were placed in a 10 μL glass chamber and sealed with a glass slide on top. The fluctuation of the fluorescent tracer particles was measured using an inverted fluorescence microscope (Axio Observer D1, Zeiss, Kohen, Germany), equipped with a Fluar 100×, N.A. 1.3, oil-immersion lens combined with a 1× optovar magnification changer.

### 2.6. Determination of Enzyme Activity

For spectrophotometric determination of enzymatic activity 0.5–5 μM enzyme was used in a KP_i_ buffer (100 mM K_2_HPO_4_/KH_2_PO_4_ pH 7.5) containing 5 mM substrate and 300 μM NADPH. The enzyme was used to start the reaction. The decrease of fluorescence of NADPH at 340 nm was measured via a Synergy™ H1 Hybrid reader from BioTek (Winooski, VT, USA) in a 96-well plate.

An Agilent 1260 series high performance liquid chromatography (HPLC) equipped with a Diode Array Detector and a Lux 3 μm Cellulose-1 (150 × 2.00 mm) chiral column (Phenomenex) was used for HPLC analyses. For determination of enzymatic activity, a reaction mixture containing 5 mM imine substrate, 100 mM glucose, 1 mM NADP^+^, in 100 mM KP_i_-buffer supplemented with 1 mM MgCl_2_ was used and 5 μM of GF3546-ST and an excess of 10 μM GDH-His for NADPH-regeneration was added. Subsequently 150 μL samples at different time points were quenched with 50 μL of 1 M NaOH saturated with NaCl. The aqueous solution was extracted with 600 μL n-heptane and 3 μL of the organic phase were injected onto HPLC for analysis. Resulting conversion rates were analyzed and calculated based on a calibration using commercially available educt/product or the racemic reduction products **3a**/**3b** of 6,7-dimethoxy-1-methyl-3,4-dihydroisoquinoline **3**. Based on the literature described (*S*)-enantioselectivity of GF3546 [19], we assumed that the formed product **3a** was (*S*)-configured. The following HPLC methods were used for substrates **2** (Method A) and **3** (Method B).

A: 97:3 (n-heptane:isopropanol) 0.5 mL/min 30 °C;
B: 93:7 (n-heptane:isopropanol) 0.5 mL/min 30 °C.

The following retention times were observed: 3,4-Dihydroisoquinoline **2**: 2.9 min; 1,2,3,4-Tetrahydroisoquinoline **2a**: 4.8 min; 6,7-dimethoxy-1-methyl-3,4-dihydroisoquinoline **3**: 3.4 min; (*S*)-1-Methyl-6,7-dimethoxy-1methyl-1,2,3,4-tetrahydroisoquinoline **3a**: 5.9–6.1 min; (*R*)-1-Methyl-6,7-dimethoxy-1methyl-1,2,3,4-tetrahydroisoquinoline **3b**: 7.8 min.

### 2.7. Racemic Reduction of 6,7-Dimethoxy-1-methyl-3,4-dihydroisoquinoline 3

6,7-dimethoxy-1-methyl-3,4-dihydroisoquinoline was purchased from VWR. For racemic reduction, one equivalent 6,7-dimethoxy-1-methyl-3,4-dihydroisoquinoline (>50 mg) was dissolved in water (1 mL) and two equivalents NaBH_4_ were slowly added under stirring at room temperature. Products were partitioned between water and n-heptane and the organic phase was analyzed via HPLC.

### 2.8. Hydrogel Preparation

Protein solutions of either GDH-SC/GF3546-ST to yield hydrogels, or GDH-His/GF3546-ST used as negative control, were diluted in KP_i_-Mg (100 mM KP_i_ pH 7.5, 1 mM MgCl_2_) to a final concentration of 500 μM in 100 μL. The hydrogels were polymerized for 1 h at 30 °C, 1000 rpm using a thermoshaker. Th buffer was evaporated using a 0.2 mL reaction tube with an open lid under constant centrifugation at 2200× *g* for 15–17 h at 30 °C. The samples were supplemented with 0.2 mg/mL “dragon” green fluorescent polystyrene microspheres (200 nm diameter; Bangs Laboratories, USA) for subsequent MPT analyses.

### 2.9. Microfluidic Setup and Analysis of the Hydrogels under Continuous Flow

The microfluidic reactor was prepared as previously described [17]. In brief the microreactor dimension was based on a standard microscope slide (76 × 26 mm² DIN ISO 8037-1:2003-05). Replica casting of polydimethylsiloxane (PDMS) (Sylgard 184, Dow Corning, Midland, MI, USA) was used for the upper part of the reaction channel using brass replication molds. The channel structure was 3 mm wide, 1 mm high, and 54 mm long yielding a volume of 150 μL. Through horizontal holes in the mold cannulas (Sterican, B. Braun Melsungen AG, Melsungen, Germany) the PDMS prepolymer was inserted prior to pouring. PDMS curing was conducted at 60 °C for at least 3 h.

The microreactors were placed inside an incubator (set to 30 °C), filled with 150 μL 1000 μM protein solution (GDH-SC/GF3546-ST 1:1), and incubated for 30 min. The PDMS chips were then sealed with a polyolefin foil (HJ-BIOANALYTIK GmbH, Erkelenz, Germany). Low pressure syringe pumps (neMESYS 290N) equipped with 5- or 10-mL syringes were connected to a manual switching valve connected to the reactor for continuous flow of reaction media. Substrate solutions were filled into 5–10 mL glass syringes containing 5 mM of substrate **2**, 100 mM glucose in KPi supplemented with 1 mM MgCl_2_, 0.01% (v/v) sodium azide to prevent fouling, and 1 mM NADP^+^. A Compact Positioning System rotAXYS or rotAXYS360 (CETONI, Korbussen, Germany) was used to allow for automatic fractioning into 96-well plates, previously loaded with 50 μL 1 M NaOH to stop the activity of possible flushed out enzymes. A CETONI neMESYS Base module, which was controlled by the QmixElements Software (CETONI GmbH, Korbussen, Germany) was connected to the syringes and sampling unit. The chip was connected to the system by conventional polytetrafluoroethylene (PTFE) tubings (ID = 0.5 mm).

## 3. Results and Discussion

### 3.1. Construction and Characterization of the All-Enzyme Hydrogels

**Scheme 1:**
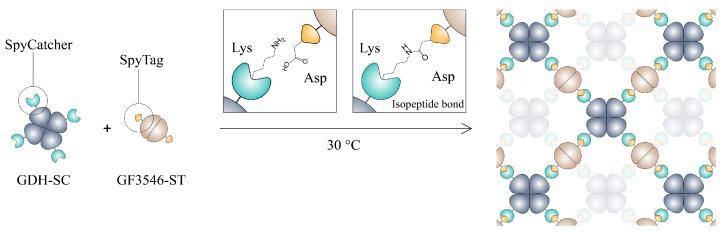
The SpyCatcher (SC)/SpyTag (ST) labelled enzymes GDH-SC and GF3546-ST spontaneously polymerize upon mixing under physiological conditions by formation of a covalent isopeptide bond between the activated lysin residue of the SC and the aspartic acid residue on the ST. This leads to formation of a porous network with alternating arrangement of the two tagged enzymes.

For the construction of a cofactor regenerating imine reductase hydrogel we used the homotetrameric glucose-1-dehydrogenase from *Bacillus subitilis* (GDH) and the homodimeric imine reductase from *Streptomyces* sp. (GF3546) [19,26]. To facilitate conjugation of the two enzymes, the previously described GDH-SpyCatcher (GDH-SC) [18] construct was combined with a GF3546-ST (SpyTag) fusion that was newly designed. The versatile SC/ST crosslinking system, originating from *Streptococcus pyogenes,* allows specific crosslinking under physiological conditions of fused proteins through the formation of an isopeptide bond between an activated lysin residue on the SC and aspartic acid residue on the ST [16]. Recombinant expression in *E. coli* and purification via metal-affinity chromatography yielded the tagged enzyme variants as pure proteins (Figure S1). Owing to their inherent oligomeric quaternary structure the SC/ST labelled GDH-SC and GF3546-ST fusion enzymes generate a covalent interconnected porous network upon mixing (Scheme 1).

**Figure 1:**
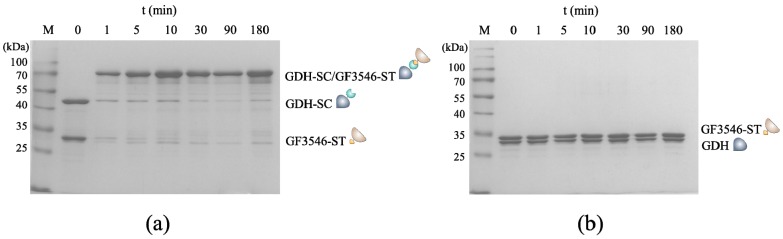
Kinetic analysis of the ST/SC-mediated conjugation of equimolar amounts (35 pmol) of GF3546-ST with (**a**) GDH-SC or (**b**) GDH, respectively. (Coomassie stained 12% SDS-PAGE gels, M: Color Pre-stained Protein Standard).

To confirm the functionality of ST- and SC-tags of the fusion enzymes, kinetic analysis of the crosslinking reaction was done by SDS-PAGE analysis using equimolar amount of GDH-SC/GF3546-ST (Figure 1a) or, as negative control, GDH/GF3546-ST (Figure 1b), respectively. The mixtures were analyzed over a course of 3 h. The SDS-PAGE analysis revealed that a new band at about 70 kDa, corresponding to the crosslinked GDH/GF3546 monomers, appeared only when both enzymes were tagged. Notably, the reaction was already completed after a few minutes after mixing.

For further characterization of the gelation process the increase in average hydrodynamic particle size of the two SC/ST labelled enzymes was determined by dynamic light scattering (DLS) analysis (Figure S2). We found that the equimolar mixture of GDH-SC/GF3546-ST showed an increase of the hydrodynamic particle diameter to a critical size regime of about 40 nm (Figure S2a). Furthermore, the viscoelastic properties of the formed hydrogel were analyzed by optical microrheology based on multiple particle tracking (MPT) (Figure S2b) [17,27]. MTP analysis revealed a G_0_ value for the GDH-SC/GF3546-ST hydrogel of 13 ± 1 resulting in a calculated mesh size of 67 ± 2 nm. These values are in good agreement with our previously published (*R*)- and (*S*)- ketoreductase based all-enzyme materials (60 and 42 nm, respectively) [17,18] and the pore size is in the range of conventional ultrafiltration membranes.

### 3.2. Determination of Catalytic Activity and Microfluidic Experiments

To investigate the biocatalytic performance of the GF3546-ST construct, conversion of three different cyclic imine substrates (**1**–**3**, in Table 1) was determined by UV-vis spectrophotometry and HPLC (Table 1, Figures S3 and S4).

We found that all substrates were accepted and converted by the enzyme to the corresponding secondary amine. In comparison with literature described WT GF3546, our His-ST fusion protein retained 55% of its activity in the conversion of substrate **1** [19]. The high stereoselectivity reported for GF3546-ST [19] was confirmed by analyzing the reaction products from the conversion of the prochiral imine **3** and, indeed, an excellent enantiomeric excess of >99% was found. Since the efficiency of enzymatic conversion was greatest in the case of **2**, all further microfluidic experiments were performed with this substrate.

**Figure 2:**
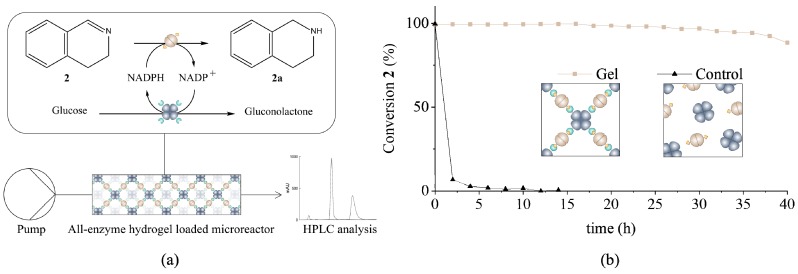
(**a**) Schematic representation of the microfluidic setup and cascade reaction in the microreactor. Imine **2** is reduced to the corresponding amine **2a** by the GF3546-ST enzyme under consumption of nicotinamide adenine dinucleotide phosphate (NADPH). The cofactor is regenerated by the GDH-SC through oxidation of glucose to gluconolactone. (**b**) Time dependent conversion of imine **2** using the microreactor filled with the all-enzyme hydrogel (squares) or the control (triangles). Substrate and cofactor were added continuously at a concentration of 5 and 1 mM, respectively, at a flow rate of 10 μL/min.

To investigate the applicability of the cofactor regenerating imine reductase hydrogel for flow biocatalysis (Figure 2), we used a microfluidic setup. Syringe pumps were used to perfuse a substrate mix containing NADP^+^, glucose, and substrate **2** through a hydrogel-loaded linear polydimethylsiloxane (PDMS) microreactor channel (*V* = 150 μL) with a flow rate of 10 μL/min (Figure 2a), which allows laminar flow profiles inside the reactor. The GDH-SC component in the gel functions as a regeneration system to recycle the expensive cofactor NADPH by reducing NADP^+^ while oxidizing glucose to gluconolactone. The outflow of the microreactor was analyzed via HPLC. (Figure S3). It is shown in Figure 2b that the GDH-SC/GF3546-ST hydrogel effectively retained the immobilized enzymes and displayed stable conversion rates of **2** over a time period of >40 h with only slight activity drops at around 35 h, which may be due to inactivation of the imine reductase. The control containing the untagged GDH was rapidly flushed out of the reactor in less than 3 h (Figure 2b).

**Figure 3:**
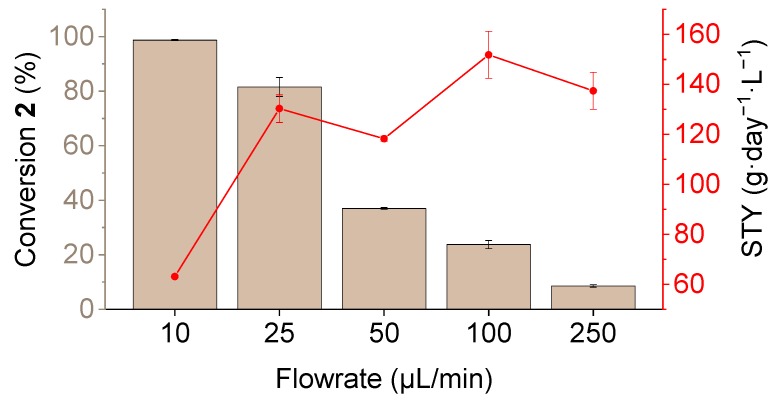
Flowrate dependent conversion of substrate **2** in the hydrogel loaded microreactor. The red curve indicates the calculated space-time yield (STY) for each flowrate. Substrate and cofactor were added continuously at 5 and 1 mM, respectively.

The productivity of the hydrogel loaded microreactor was further analyzed under variable flow rate conditions (Figure 3). Space-time yields (STY) were calculated on basis of the reactor volume of 150 μL and the conversion rate of substrate **2** at the different flow rates as determined from HPLC analyses. Variation of the flowrate from 10 to 250 μL/min and calculation of the corresponding STYs indicated that the largest productivity was reached at 100 μL/min with about 150 g·(L·day)^−1^ in the PDMS reactor. By optimizing the reactor structure via increasing the surface to volume ratio even higher STYs should be achievable.

## 4. Conclusions

We successfully constructed and applied a cofactor regenerating imine reductase hydrogel consisting of only SC/ST labelled GDH and GF3546. The novel material clearly expands the scope of our all-enzyme hydrogel concept, since it enables the reduction of imines to be facilitated readily in a continuous microfluidic process. To the best of our knowledge no imine reductases were yet implemented into a continuous flow system. However, work on an artificial imine reductase immobilized on silica nanoparticles [22] and a NADH accepting imine reductases on controlled porous glass [23] has been reported. In the latter case, the purified WT enzyme remained about 50% of initial activity after the first cycle of catalysis (22 h).

The reactors presented here showed stable conversion rates for up to 40 h and a maximum STY of 150 g·(L·day)^−1^ at a flow rate of 100 μL/min. These results emphasize that that our self-assembling hydrogel approach is an efficient means for mild and effective immobilization which can be applied to various other enzyme classes like e.g., decarboxylases, reductive aminases, or transaminases. The preparation of the all-enzyme hydrogels might even be possible from crude cell lysates in order to simplify the formulation in terms of time and cost effectiveness. Additionally, future work aims to systematically benchmark our hydrogel methodology against chemical cross-linked or particle-based immobilization techniques. We believe that our novel technology holds great potential to yield a versatile toolbox for enzymatic cascade reactions under continuous flow conditions.

## Figures and Tables

**Table 1:** Biocatalytic activity on different cyclic imine substrates of the GF3546-ST enzyme. All enzymatic activities were tested at a substrate concentration of 5 mM.

Substrate	Specific Activity (μmols_ubstrate_·(μmol_enzyme_·min)^−1^)	Ee (%)
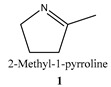	2.4 ^a^ ± 0.4	n. d.
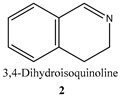	83.3 ^a^ ± 8.2	-
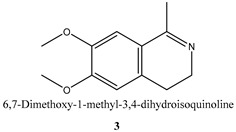	2.3 ± 0.3 ^b^	>99 ^b^

^a^ Determined by UV-Vis spectrophotometry; ^b^ Determined by HPLC. All measurements were performed as triplicates.

## Supplementary Materials

The following are available online at https://www.mdpi.com/2072-666X/10/11/783/s1, Figure S1: SDS-gel electrophoretic analysis of purified proteins used in this study. Figure S2: Dynamic Light Scattering (DLS) and optical microrheology measurements of the GDH-SC/GF3546-ST gels. Figure S3: Representative HPLC traces of 3,4-Dihydroisoquinoline **2** and the corresponding Amine **2a**. Figure S4: Representative HPLC traces of 6,7-Dimethoxy-1-methyl-3,4-dihydroisoquinoline **3** and the corresponding Amines **3a** and **3b**.
