# Supplementary material for: Imine Reductase Based All-Enzyme Hydrogel with Intrinsic Cofactor Regeneration for Flow Biocatalysis
Source: Micromachines (Basel). 2019 Nov 15;10(11):783. doi: 10.3390/mi10110783 (PMC6915733; doi:10.3390/mi10110783)
Supplement: Supplementary file 1 [file micromachines-10-00783-s001.pdf]

micromachines

MDPI

Supplementary Materials

Imine Reductase Based All-Enzyme Hydrogel with Intrinsic Cofactor Regeneration for Flow Biocatalysis

Patrick Bitterwolf, Felix Ott, Kersten S. Rabe and Christof M. Niemeyer *

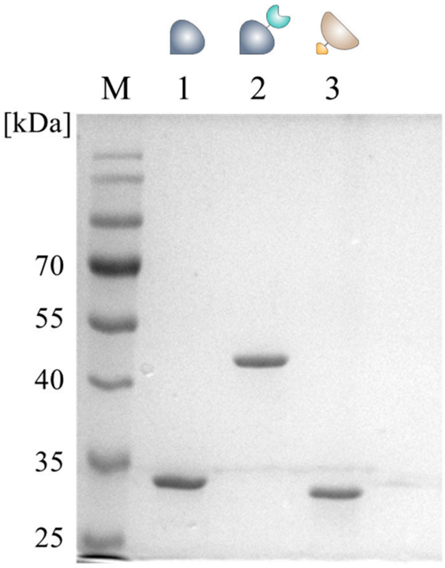

Figure S1. SDS-gel electrophoretic analysis of the purified recombinant proteins, used in this study. This is a coomassie stained 12% SDS-PAGE. M: Color Prestained Protein Standard, Broad Range (New England Biolabs); lane 1: GDH-His (29 kDa); lane 2: GDH-SC-His (41 kDa); lane 3: GF3546-ST (33 kDa).

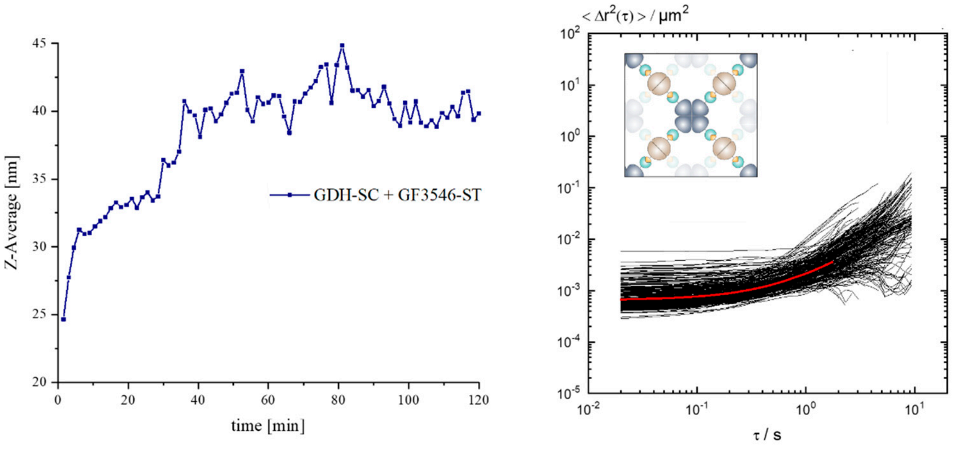

(a) (b)

Figure S2. (**a**) Time-dependent increase of the hydrodynamic particle diameter of a 1 mM equimolar mixture of GDH-SC/GF3546-ST determined by DLS. (**b**) Optical microrheology measurements based on multiple particle tracking (MTP) analyses of gelated GDH-SC/GF3546-ST. Mean square displacements (MSDs) of individual polystyrene microspheres of 200 nm diameter determined for the gelated GDH-SC/GF3546-ST material were recorded (black curves). The red curve shows the ensemble-average MSD. Note that the MSDs exhibit almost no time dependence indicating that tracer particles are highly constrained by the surrounding hydrogel.

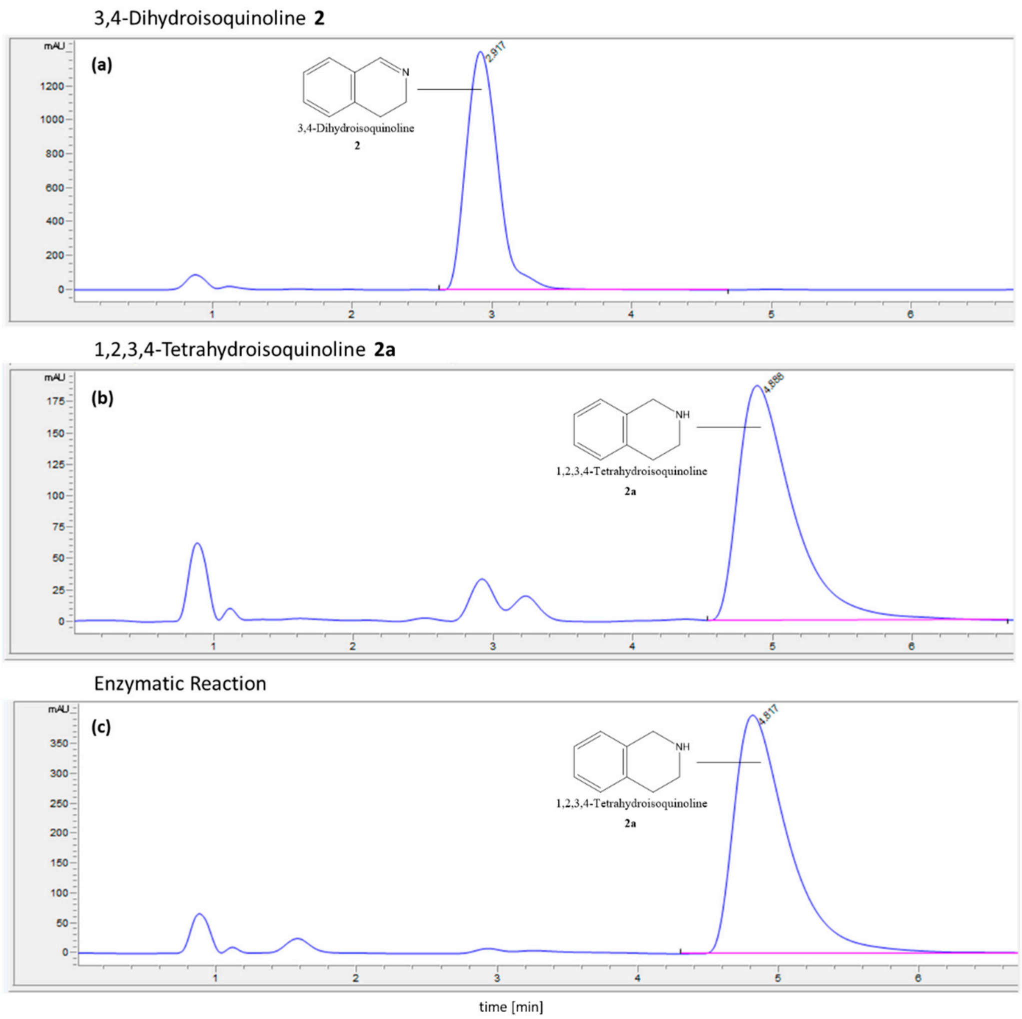

Figure S3. Representative HPLC traces of (**a**) the educt 3,4-Dihydroisoquinoline **2** and the corresponding amine product obtained from (**b**) chemical or c) enzymatic reduction by GF3546-ST.

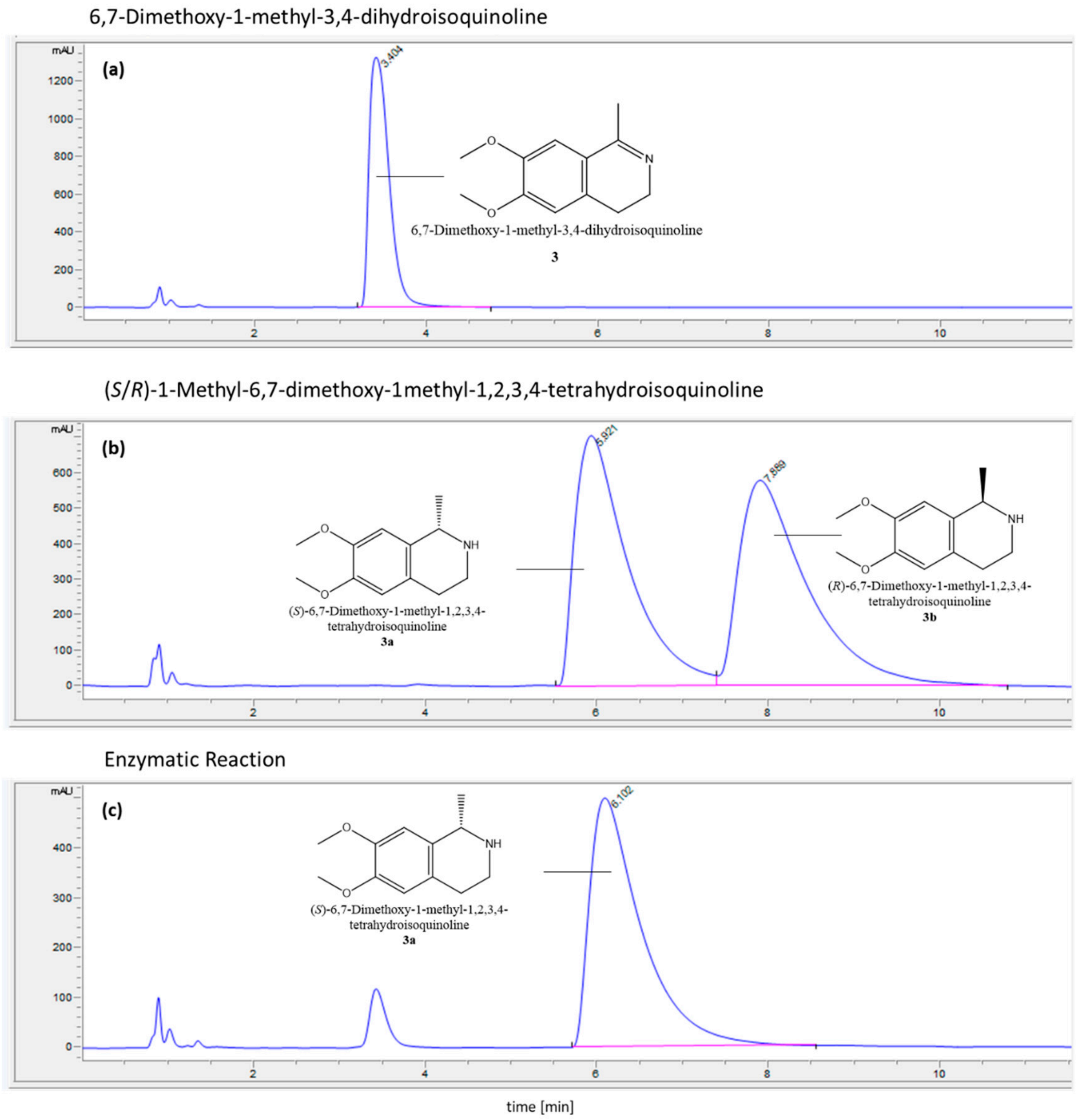

Figure S4. Representative HPLC traces of (**a**) the educt 6,7-Dimethoxy-1-methyl-3,4-dihydroisoquinoline **3** and the corresponding amine product obtained from (**b**) chemical or (**c**) enzymatic reduction by GF3546-ST.

© 2019 by the authors. Submitted for possible open access publication under the terms and conditions of the Creative Commons Attribution (CC BY) license (http://creativecommons.org/licenses/by/4.0/).
